# Applying A/B Testing to Clinical Decision Support: Rapid Randomized Controlled Trials

**DOI:** 10.2196/16651

**Published:** 2021-04-09

**Authors:** Jonathan Austrian, Felicia Mendoza, Adam Szerencsy, Lucille Fenelon, Leora I Horwitz, Simon Jones, Masha Kuznetsova, Devin M Mann

**Affiliations:** 1 Department of Medicine NYU Grossman School of Medicine New York, NY United States; 2 Medical Center Information Technology NYU Langone Health New York, NY United States; 3 Department of Population Health NYU Grossman School of Medicine New York, NY United States

**Keywords:** AB testing, randomized controlled trials, clinical decision support, clinical informatics, usability, alert fatigue

## Abstract

**Background:**

Clinical decision support (CDS) is a valuable feature of electronic health records (EHRs) designed to improve quality and safety. However, due to the complexities of system design and inconsistent results, CDS tools may inadvertently increase alert fatigue and contribute to physician burnout. A/B testing, or rapid-cycle randomized tests, is a useful method that can be applied to the EHR in order to rapidly understand and iteratively improve design choices embedded within CDS tools.

**Objective:**

This paper describes how rapid randomized controlled trials (RCTs) embedded within EHRs can be used to quickly ascertain the superiority of potential CDS design changes to improve their usability, reduce alert fatigue, and promote quality of care.

**Methods:**

A multistep process combining tools from user-centered design, A/B testing, and implementation science was used to understand, ideate, prototype, test, analyze, and improve each candidate CDS. CDS engagement metrics (alert views, acceptance rates) were used to evaluate which CDS version is superior.

**Results:**

To demonstrate the impact of the process, 2 experiments are highlighted. First, after multiple rounds of usability testing, a revised CDS influenza alert was tested against usual care CDS in a rapid (~6 weeks) RCT. The new alert text resulted in minimal impact on reducing firings per patients per day, but this failure triggered another round of review that identified key technical improvements (ie, removal of dismissal button and firings in procedural areas) that led to a dramatic decrease in firings per patient per day (23.1 to 7.3). In the second experiment, the process was used to test 3 versions (financial, quality, regulatory) of text supporting tobacco cessation alerts as well as 3 supporting images. Based on 3 rounds of RCTs, there was no significant difference in acceptance rates based on the framing of the messages or addition of images.

**Conclusions:**

These experiments support the potential for this new process to rapidly develop, deploy, and rigorously evaluate CDS within an EHR. We also identified important considerations in applying these methods. This approach may be an important tool for improving the impact of and experience with CDS.

**Trial Registration:**

Flu alert trial: ClinicalTrials.gov NCT03415425; https://clinicaltrials.gov/ct2/show/NCT03415425. Tobacco alert trial: ClinicalTrials.gov NCT03714191; https://clinicaltrials.gov/ct2/show/NCT03714191

## Introduction

Clinical decision support (CDS) is a valuable feature of electronic health records (EHRs). CDS can generate several forms of decision support that have been extensively studied, including alerts, calculators, reminders, and order sets [1-3]. Successful examples of CDS have led to reductions in prescribing brand-name antibiotics [4], improved lipid management in renal transplant patients [5], improved compliance with guidelines for treating HIV [6-8], reduced ordering of tests when costs were displayed [9], and age-specific alerts that reduced inappropriate prescribing in the elderly [10-15]. Although conceptually straightforward, successful CDS alerts must deliver accurate information, in clinical context and at the point of care, and must be well-integrated into the clinical workflow. These complexities have contributed to inconsistent results [16,17] that reflect the enormous heterogeneity in system design, workflow integration, usability, simplicity, and content [18,19].

Despite the complexity of designing effective CDS and the inconsistent results, the sheer volume of CDS in modern EHRs has increased dramatically. In the past 5 years, the number of alerts released in our institution’s EHR increased from 13 to 117. This volume has amplified the underlying challenges of clunky user interfaces, poor usability, and workflow integration and has fueled the rapid escalation of alert fatigue [20-22]. Alert fatigue has yielded an environment in which providers ignore clinical alerts at rates as high as 70% of the time or more [23].

Consequently, while many CDS have been effective in promoting better care, they do so with generally poor efficiency. Moreover, EHRs burden providers with tedious, cognitively draining documentation requirements, leaving little time or energy to engage with CDS tools that have the potential to improve care but are not necessary requirements to complete a visit [24]. Alert fatigue contributes to provider burnout, a highly publicized phenomenon that is a major threat to patient safety and physician satisfaction.

CDS designers’ capabilities are often constrained by the limitations of commercial EHR systems and the operational challenges inherent to local customizations; new CDS tools are frequently released with minimal modification of the standard tools. Similarly, traditional randomized controlled trials (RCTs) require a long, static experimental design to determine the potential statistical advantage of the intervention. These approaches stand in contrast to how other industries launch new products and determine their value. Software development and other industries leverage rapid experimentation processes, often termed “A/B trials,” to efficiently evaluate multiple design choices using live environments and users [25]. In this paradigm, there is an a priori acknowledgement that the “best” version of the product is unknown until it is empirically tested. This approach is applied to big and small design choices, from what features to include in a new smartphone app to where to place icons on a web page. To enable this process, agile procedures have been developed to quickly implement versions, assess the relative impact, and then modify the product for the next round of A/B testing. Highly agile companies like Amazon use this rapid experimentation to deliver the optimal customer experience and release code modifications every 11.7 seconds to accommodate their rapid learning [26]. We believe CDS developers can deploy A/B testing methods to evaluate many aspects of evaluation including promotion of positive outcomes, minimizing unintended consequences on safety, and poor user experience.

The inpatient influenza alert was intended to promote vaccination for eligible patients. The interruptive alert triggered at the time the nurse documented responses to the influenza screening question flowsheets. Acceptance was defined as placing the order for the influenza vaccine. The alert continued to trigger from flowsheet documentation until the patient was ordered for the vaccine or the patient no longer met eligibility criteria.

The outpatient tobacco cessation alert was designed to promote counseling and treatment for patients using tobacco products. The noninterruptive alert displayed in an alert section for outpatient providers at chart opening when active smokers had not received counseling within the previous 3 months. Acceptance was defined as documentation of counseling, prescription of therapy, or referral to the state tobacco quit line. We hypothesized that A/B testing methods would enable our CDS development teams to quickly evaluate CDS designs and iteratively modify them to maximize their acceptance rates and impact on clinical outcomes while minimizing their firing rates.

## Methods

### Creating an A/B Testing Framework

**Figure 1:**
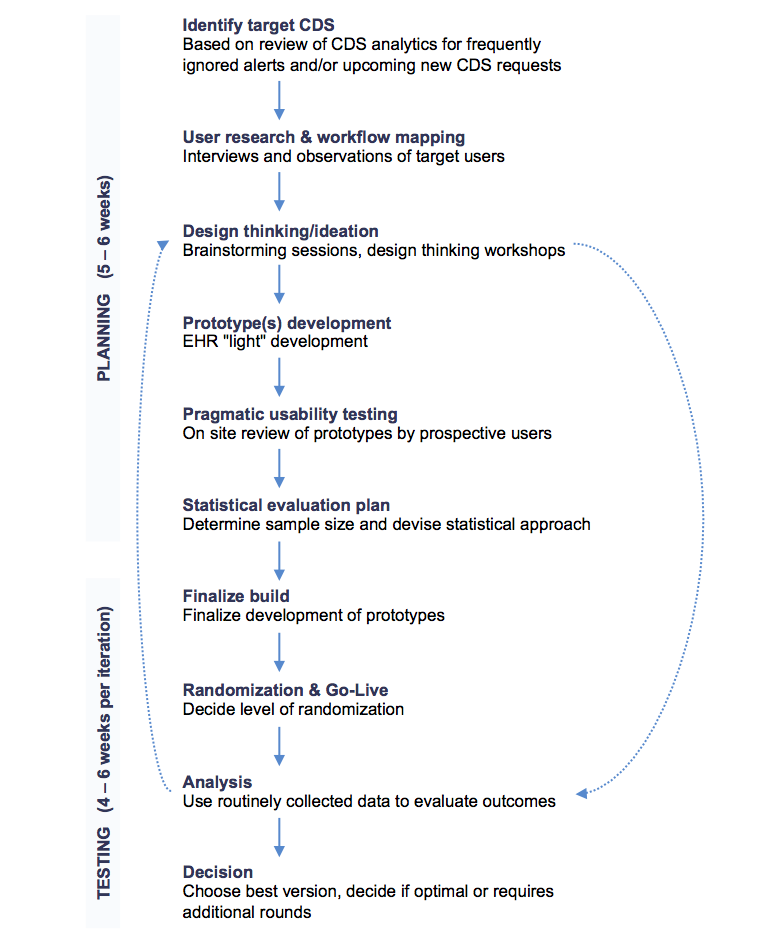
Clinical decision support (CDS)/randomized controlled trial (RCT) process map. EHR: electronic health record.

The CDS optimization initiative was a partnership between our CDS team and the Rapid RCT lab at NYU Langone Health [27]. The Rapid RCT lab uses elements of the traditional RCT methodology in a series of rapid cycle experiments to test and optimize systems interventions at our institution. Together, the CDS and Rapid RCT lab teams collaboratively defined a new process combining A/B methods with traditional RCTs to optimize our CDS alerts. To achieve this synergy, a multidisciplinary team was assembled including physician and nurse informaticists, implementation scientists, EHR analysts, data analyst, statistician, project manager, and project associate. A multistep process was developed that combined tools from user-centered design, A/B testing, and implementation science (Figure 1).

This process was supported by 3 key “enablers.” One was the work done by the Rapid RCT lab to engage the local institutional review board (IRB) and create sufficient understanding of how A/B testing works so that these projects would typically be self-certified as quality improvement research and not require IRB review. This enabled our team to develop and test each iteration of a CDS alert at a rapid pace. Second, our CDS team engaged the relevant health system leadership early in the process for each alert so that there was sufficient buy-in for this novel approach to CDS development and optimization. This required multiple presentations on our process to develop trust in how it would improve the clinician experience with CDS and acceptance that there would be multiple versions of each CDS, creating heterogeneity in the user experience. This effort has been ongoing and critical to educating clinicians and administrators about this highly novel work that touches many important clinical processes. Third, our CDS team developed its technical ability to randomize experiences in the EHR at the patient level (Multimedia Appendix 1). This work leveraged native EHR functionality. However, this logic did not provide for automated randomization at the practice level. To overcome this limitation, our team developed a process for manual randomization of clinical practices and then created logic to assign the appropriate CDS version to the appropriate target group. Fourth, we created a robust ability to report on CDS tool engagement that gave us near real-time access to the impact of each new version. Our team developed reporting capabilities beyond the standard EHR tools to drill down on what parts of each CDS tool were being “clicked” versus “ignored,” what orders were being placed from what exact location, and trends in utilization. These tools allowed the team to quickly ascertain if new trends were emerging. Last, we prespecified safety metrics before each go-live. Along with our CDS outcomes, balancing clinical outcomes were evaluated to track performance and ensure patient quality and safety after each iteration. These capabilities unlocked our team’s ability to scale rapid, randomized CDS experiments.

### Application of the Framework

Based on the A/B testing process described in the previous section, our team was now positioned to implement our CDS A/B testing process more widely across our enterprise. We use 2 examples, our inpatient, nurse-facing influenza vaccine alert and our ambulatory, provider-facing tobacco cessation counseling alert, to detail the experimental methods involved in implementing our CDS A/B testing process.

The first step involved rapid user research. Both of these alerts were already active, with low rates of acceptance by the relevant users: 2% for the influenza vaccine alert and 31% for the tobacco cessation alert. Our process involved conducting interviews and observations (usually by our clinician informaticists and a research team member) of users interacting with the alerts using our pragmatic usability testing method to quickly summarize field notes that could be reviewed by the design team [28]. With these data in hand, the team conducted ideation sessions to develop potential alternatives to the current CDS that could enable higher adoption rates. These included modifications based on CDS best practices to reduce cognitive load (embedding images, simplifying text, consistency of formatting) [29-31] and other motivational tools (“nudges,” such as highlighting financial incentives [eg, relative value units] or using more authoritative messaging) [32].

Once the team identified the versions of interest, the EHR development team created lightweight (eg, paper-based illustrations) prototypes of each version for further review by stakeholders. After iterative rounds of refinement, the prototypes were fully built within our EHR testing environment for additional usability testing and feedback from target users. With these data, the multiple CDS versions were then built and randomized to control for unmeasured confounders (eg, changes in patient volumes, staffing changes, seasonal changes), and deployed into the live environment. The inpatient influenza alert and the tobacco alert were randomized at the patient and practice level, respectively.

### Statistical Analysis

Once deployed, our team used relevant CDS metrics (alert views and follow-up actions such as placing a suggested order) and the predetermined statistical approach to evaluate which CDS version was superior. Initially, we used only simple statistics (*t* tests or Chi square tests) to compare outcomes between versions. Once the testing cycle was completed, the team reconvened and evaluated if the improvement (or lack thereof) was sufficient or if additional rounds of A/B testing were warranted.

Post-hoc analysis initiated based on result irregularities in the cluster randomization tobacco experiment revealed unanticipated behaviors in the 3 groups that resulted in different baseline rates by randomization group. To control for these underlying irregularities, we modified our methods to employ a more sophisticated analysis. A multilevel logistic regression model was developed to predict alert acceptance, using the lme4 library from R [33]. Ambulatory practice was included as a random effect, and randomization group and study rounds were included as fixed effects. This method produced odds ratios that compared acceptance rates of different alert modification groups to a reference group. We obtained odds ratios from the models and profiled confidence intervals for the odds ratios using the approach of Venables and Ripley [34].

## Results

### Flu Alert Experiment

**Figure 2:**
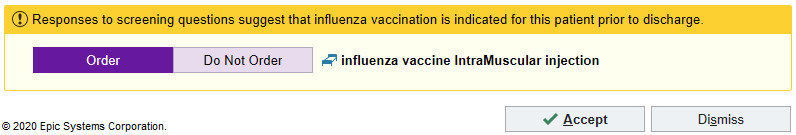
Original version of the flu alert tested in the randomized controlled trial (RCT). Copyright 2020, Epic Systems Corporation.

**Figure 3:**
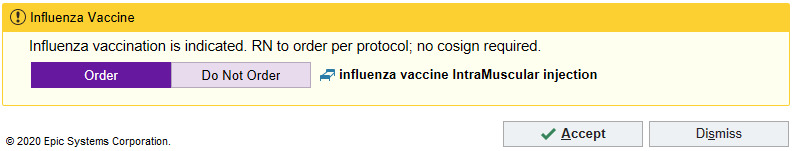
New version of the flu alert tested in the randomized controlled trial (RCT), with a simpler header and more directed verbiage that states “RN to order. Per Protocol; no cosign required”. Copyright 2020, Epic Systems Corporation.

Our week-long user feedback sessions highlighted several issues, including confusion about the need for the ordering nurse to obtain a physician cosignature and difficulty of appropriately documenting patient refusal to prevent future triggering of the alert. This feedback was used to tailor the alert to the appropriate setting and user. We first conducted a 5-week RCT to address the most frequently mentioned nursing barrier: a misconception that ordering the influenza vaccine was outside of the nurse’s scope of practice. We tested whether explicitly stating nurses were empowered to order the vaccine with no cosignature requirement would improve alert acceptance. We randomized patients into 2 arms: the existing alert (Figure 2) and the new alert that stated that nurses can order the vaccine without cosignature (Figure 3). The new message resulted in a negligible reduction in firings per patient per day.

**Figure 4:**
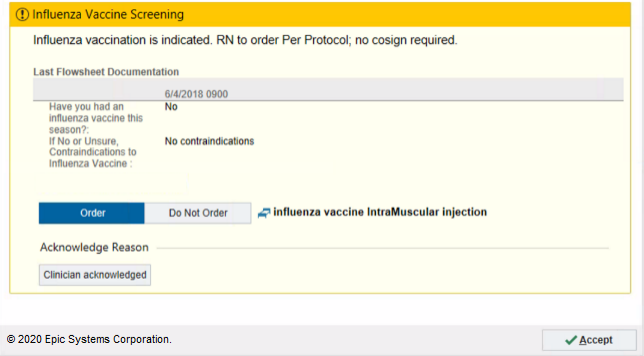
Version 3 of the flu alert, in which an acknowledgement reason button was added and the “dismiss” button was removed. Copyright 2020, Epic Systems Corporation.

This limited impact encouraged the team to conduct a more intensive review of the triggering logic and user response options. This review had surfaced fundamental flaws inherent in both versions of the alert. These enhancements were prioritized, and the change most likely to succeed was implemented in version 3. Specifically, the team identified that nurses did not appreciate that when they dismissed the alert with no action, the alert would appear automatically again at next flowsheet filing. Consequently, for version 3, we eliminated the ability to “dismiss” the alert without ordering the vaccine or selecting the acknowledgment reason. When nurses attempted to rapidly satisfy the alert with “accept,” the influenza order would automatically be placed, satisfying the alert and suppressing future firings (Figure 4). The CDS design team concluded that testing a new alert against an original version in a rapid RCT was no longer justifiable as we did not believe the 2 options had equipoise and did not want to delay enhancements that had high likelihood of reducing nurse fatigue. Version 3 resulted in a 64% reduction in firings per patient per day (Table 1).

**Table 1:** Flu alert results.

Round	Alert version	Firings per patient per day	*P* value	Vaccination compliance rate at discharge, %
Baseline (Nov 2018 - Jan 2018)	1 (n=8296)	23.0	N/A^a^	N/A
1 (Feb 2018 - Apr 2018)	1 (n=2025)	23.6	.521^b^	90.8
1 (Feb 2018 - Apr 2018)	2 (n=2039)	23.1		
2 (Sep 2018 - Dec 2018)	3 (n=8777)	8.4	<.001^c^	87.7
3 (Jan 2019 - Feb 2019)	4 (n=1952)	7.3	<.001^c^	89.0

^a^N/A: not applicable.

^b^Comparing 1 vs 2 in the randomized controlled trial.

^c^Compared to baseline.

**Figure 5:**
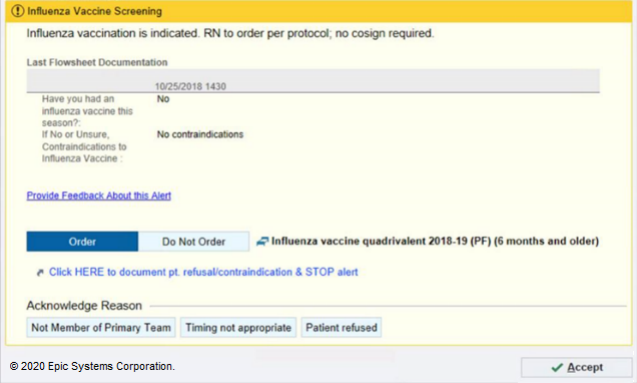
Version 4 of the flu alert, in which new acknowledgement reason buttons with lockout periods and a jumplink to update flowsheet documentation was added, and inappropriate units were excluded. Copyright 2020, Epic Systems Corporation.

The CDS design team implemented the next prioritized enhancements in version 4. For the fourth cycle, the team identified that nurses disproportionally dismissed alerts in specific hospital units, including the Post-Anesthesia Care Unit. Upon learning about this finding, nursing leadership determined that these units were not appropriate locations to trigger the alert. Furthermore, our usability testing identified that the alert did not offer nurses sufficient locus of control. Specifically, nurses could not suppress the alert for a period of time when they felt the timing or recipient of the alert was inappropriate without placing the order. Consequently, we added targeted acknowledgement buttons to the alert based on these reasons that prevented the alert from firing for an acceptable period of time (Figure 5). This improvement resulted in a 13% reduction in alert firings per patient per day compared to version 3 (Table 1). These mixed methods served as a reminder that while A/B testing is a helpful tool, employing A/B testing in all situations is not the goal.

### Tobacco Alert Experiment

**Figure 6:**
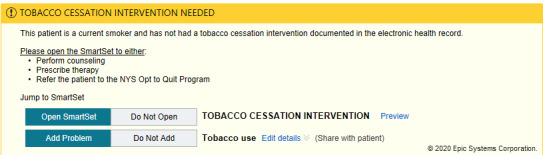
Baseline tobacco cessation alert. Copyright 2020, Epic Systems Corporation.

**Figure 7:**
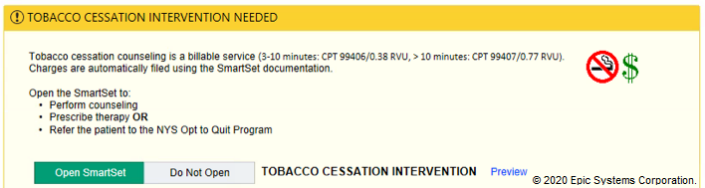
Tobacco financial messaging alert with images tested in Round 2 of the randomized controlled trial. Copyright 2020, Epic Systems Corporation.

**Figure 8:**
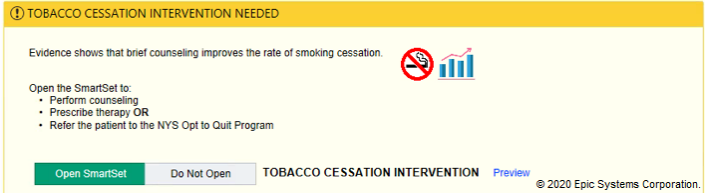
Tobacco evidence-based messaging alert with images tested in Round 2 of the randomized controlled trial. Copyright 2020, Epic Systems Corporation.

**Figure 9:**
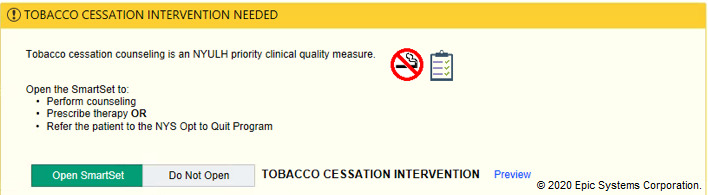
Tobacco regulatory messaging alert with images tested in Round 2 of the randomized controlled trial. Copyright 2020, Epic Systems Corporation.

**Figure 10:**
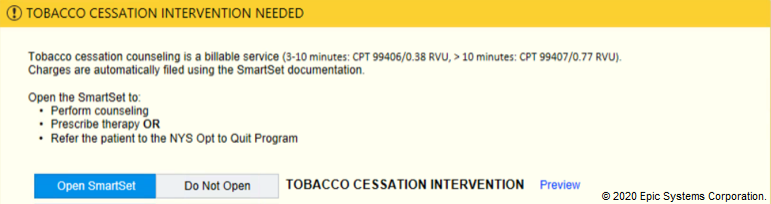
Tobacco financial messaging alert with no images tested in Round 3 of the randomized controlled trial. Copyright 2020, Epic Systems Corporation.

**Figure 11:**
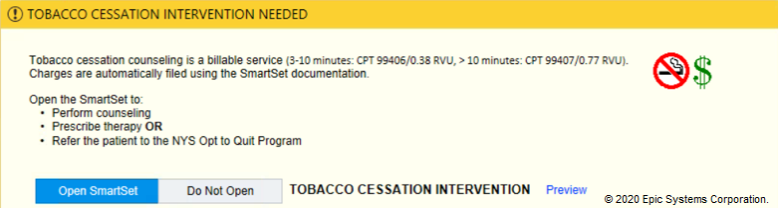
Tobacco financial messaging alert with both images (no smoking sign and dollar sign) tested in Round 3 of the randomized controlled trial. Copyright 2020, Epic Systems Corporation.

**Figure 12:**
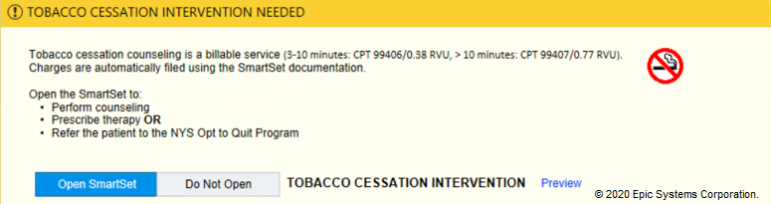
Tobacco financial messaging alert with image of no smoking sign tested in Round 3 of the randomized controlled trial. Copyright 2020, Epic Systems Corporation.

In the tobacco alert experiment, our week-long design thinking exercises produced 3 potential improvements with varying message framing (financial, evidence-based, regulatory) and complementary images (Figures 6-12). A financial framing indicated the additional revenue that a physician could generate by performing tobacco cessation counseling and gave them tools to document appropriately and create the relevant billing charge. The evidence framing highlighted that tobacco cessation was a part of providing high-quality care, and the regulatory framing indicated that tobacco cessation counseling was integral to the institution’s expectations and policies. Additionally, in Round 2, images were added to reinforce the message framing.

Each version was randomized at the ambulatory practice level for 4-5 weeks. In a series of 3 A/B testing experiments over a period of 8 months, our team observed that neither the framing method nor the addition of multiple or single images resulted in significant differences in acceptance rates for the tobacco alert (Table 2).

**Table 2:** Acceptance rates by alert type (encounter level acceptance rates; acceptance includes any positive action — perform counseling, prescribe therapy, or refer to state tobacco quit line).

Randomization group	Baseline acceptance rate (N=31650), number accepted/number displayed (%)	Round 1, Oct 2018 - Nov 2018 (N=26,975), number accepted/number displayed (%)	OR^a^ (95% CI)	Round 2, Jan 2019 - Feb 2019 (N=11,631), number accepted/number displayed (%)	OR (95% CI)	Round 3, Apr 2019 - May 2019 (N=15,811), number accepted/number displayed (%)	OR (95% CI)
A	2327/8782 (26)	2045/7621 (27), financial incentive message	0.89 (0.48-1.67)	817/3335 (24), financial message with images	0.87 (0.47-1.59)	1036/4395 (24), financial message with no images	0.90 (0.49-1.65)
B	2171/10,585 (21)	1832/8821 (21), evidence-based message	0.90 (0.50-1.63)	705/3702 (19), evidence-based messages with images	0.88 (0.49-1.58)	903/4991 (18), financial message with images	0.90 (0.50-1.64)
C	2619/12,283 (21)	2682/10,533 (25), institutional priority message	1.00	1122/4594 (24), institutional priority message with images	1.00	1295/6425 (20), financial message with tobacco sign only	1.00

^a^OR: odds ratio.

## Discussion

Our experience and results provide significant insights into the opportunities and challenges in transitioning to a strategy of optimization that has worked so successfully in other areas of information technology.

By creating an infrastructure to support this approach, we successfully tested multiple versions of alerts in a short period of time with outcomes that are supported by rigorous methods. This capability stands in contrast to the common approach of releasing single versions of CDS alerts with no empiric data supporting the relative efficacy of their design and then relying on weaker pre-post statistical methods for assessment.

This success is predicated on having assembled a multidisciplinary team, institutional support, and a repeatable process that can be applied to the rapid improvement of any CDS tool. It draws heavily on both the user-centered, agile approach from software and other quality improvement philosophies and traditional RCT methodologies. In our experience, standing up this approach is the most challenging phase. It requires effort to foster buy-in from the IRB, operational leadership, enhanced EHR randomization capabilities, and reporting to rapidly test CDS variations. The creation of the process drew heavily on our prior experience in usability and user-centered design [35] and a flexible randomization schema that could be tailored to the dynamic research methodology.

While we were successful at developing this infrastructure, the outcomes from our initial experiments offer guidance to other institutions embarking on a similar approach. For our first A/B testing experiment in influenza, we intentionally chose a less invasive design change (verbiage and mild display modifications) that would not be controversial to operational leaders and minimized risk of unintended consequences in our deployment. We recommend this approach as it allowed the information technology team members and clinical members to focus their efforts on creating the robust testing infrastructure without the distraction of complex intervention changes. The first experiment confirmed that a simple solution would not result in a dramatic improvement in alert acceptance. While strategizing the next experiment, the team uncovered more fundamental design flaws with clear remedies. Rather than subjecting these remedies to a round of RCTs, we opted to follow a traditional pre-post evaluation given the extremely high likelihood of success. This approach proved successful as we had dramatic increases in alert acceptance rates. All nurses benefited from this enhancement rather than having to wait for the result of a second RCT. Consequently, CDS teams have to consider the appropriate use cases in deploying randomized A/B testing vs more traditional heuristics-based approaches to improving CDS [21,36]. In the future, A/B testing could be more beneficial after these basic heuristic constructs have been satisfied.

In addition to the right use case, there are outstanding questions related to the right volume of usability testing vs rapid A/B testing to deploy when refining the CDS. Usability testing is significantly more resource and time intensive as compared to rapid A/B testing. Consequently, we will continue to explore how to balance a priori usability testing versus empiric A/B tests.

Similarly, despite repeated cycles, no intervention improved the acceptance rate of the tobacco alert. This result is likely because clinical practice behaviors are challenging to modify and changes to CDS presentation displays might not be sufficiently impactful. While small tests of change could be helpful to large internet sites with millions of users per day, these changes might not translate as well to CDS with a smaller, highly trained population. With our growing confidence in our underlying framework and approach, we will begin to experiment with larger structural changes to our CDS that are more likely to have impact.

Despite the successes in our implementation strategy, there are several constraints to this approach. Randomizing at the clinician level continues to be a challenge as our EHR does not easily support it and manual randomization of thousands of providers is suboptimal. Moreover, due to the pragmatic nature of the research, contamination is always a risk since clinicians and clinical workflows are not static. For instance, the flu alert was randomized at the patient level, which meant that nurses likely interacted with both versions of the alert even on the same day, possibly reducing the potential effect size.

Our cluster randomization, while minimizing contamination bias, was challenging given underlying unanticipated irregularities in the practice sites. Specifically, operational reorganization of the ambulatory network during the trials distorted the original randomization. Furthermore, we assumed that randomization had successfully distributed key characteristics across arms and initially did not check baseline rates in randomized groups. We corrected for this using statistical procedures and now examine baseline rates of all key variables prior to randomization. We also did not include a baseline control arm, which made interpretation of our results more challenging. In the future, to mitigate inevitable changes in organization structures, cluster randomization should be stratified by practice groups in addition to by individual practices. Nonetheless, organizational changes may disrupt pragmatic experiments reinforcing the importance of a rapid, iterative evaluation framework.

Finally, post-hoc analysis revealed that our statistical team had misinterpreted data retrieved from the EHR. Data from each round were captured at the EHR department level. However, our analysis was conducted at the clinical practice level. This disconnect required our team to manually map EHR departments to clinical practices, which were not always in a 1:1 relationship. This allocation was further complicated by operational changes where practices were removed and combined. For the future, we would recommend randomizing at the level data will be reported (ie, at the EHR department level for cluster randomization).

These experiences highlight the importance of having the capability to make quick, data-driven decisions and a process to rapidly remediate mistakes and apply these learnings to future A/B cycles.

Fundamental differences between health care organizations and software companies provide additional challenges to prioritizing A/B experiments. Unlike large software companies whose primary key performance indicators are dependent on user acceptance of decision support (eg, clicking on an advertisement), alert burden is not yet the major priority of clinical institutions, though it is gaining importance given the rising appreciation of physician burnout [37].

Rapid A/B testing of CDS alerts in combination with RCT methods is a promising approach to efficiently, rapidly, and rigorously evaluating the impact of the tools and the clinicians’ experience using them. Our experience also highlights the evaluative challenges associated with cluster randomization and the outstanding need for collaboration with EHR vendors to design scalable randomization approaches at various levels. Applied broadly, this approach could also help reduce the amount of CDS “noise” in the system, by both reducing the number of alerts and making each more impactful. If this proves true, the application of rapid A/B testing and RCT methods to CDS alerts could be a potential intervention for alert fatigue and improve the EHR experience.

## Appendix

Multimedia Appendix 1 A tip sheet on how to randomize within Epic created by our institution's Epic Research team.

Multimedia Appendix 2 Consort-EHealth V1.6 publication submission form.

## Abbreviations

CDS: clinical decision support
EHR: electronic health record
IRB: institutional review board
RCT: randomized controlled trial
