# Supplementary material for: Applying A/B Testing to Clinical Decision Support: Rapid Randomized Controlled Trials
Source: J Med Internet Res. 2021 Apr 9;23(4):e16651. doi: 10.2196/16651 (PMC8065554; doi:10.2196/16651)
Supplement: Multimedia Appendix 1 [file jmir_v23i4e16651_app1.pdf]

Using Rule Editor and under the context of Patient BPA Locator is property **Random Number**. Creating a rule with this Epic released property and plugging it into a Criteria record will help with randomization.

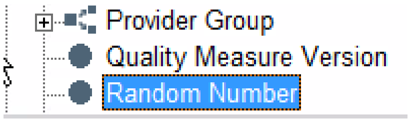


© 2020 Epic Systems Corporation.

The property in the rule should be setup to look to a whole number value and include an appropriate maximum/minimum depending on the intended frequency.

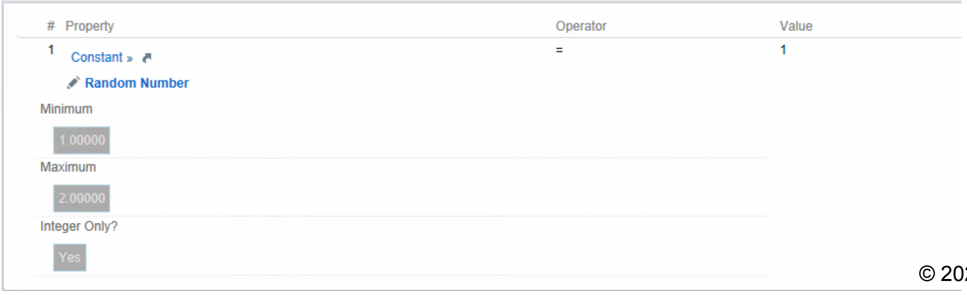


© 2020 Epic Systems Corporation.

The system will assign patient encounter contacts a random whole number and evaluate against the rule accordingly.

Adding this linked criteria to a BPA with the appropriate logic will be what sets it up as Random or not.

In our use case, we wanted to randomize when a BPA for the flu vaccine would present. We also did not want it to present in instances where another BPA or this BPA has already fired.

Our general BPA setup followed the below.

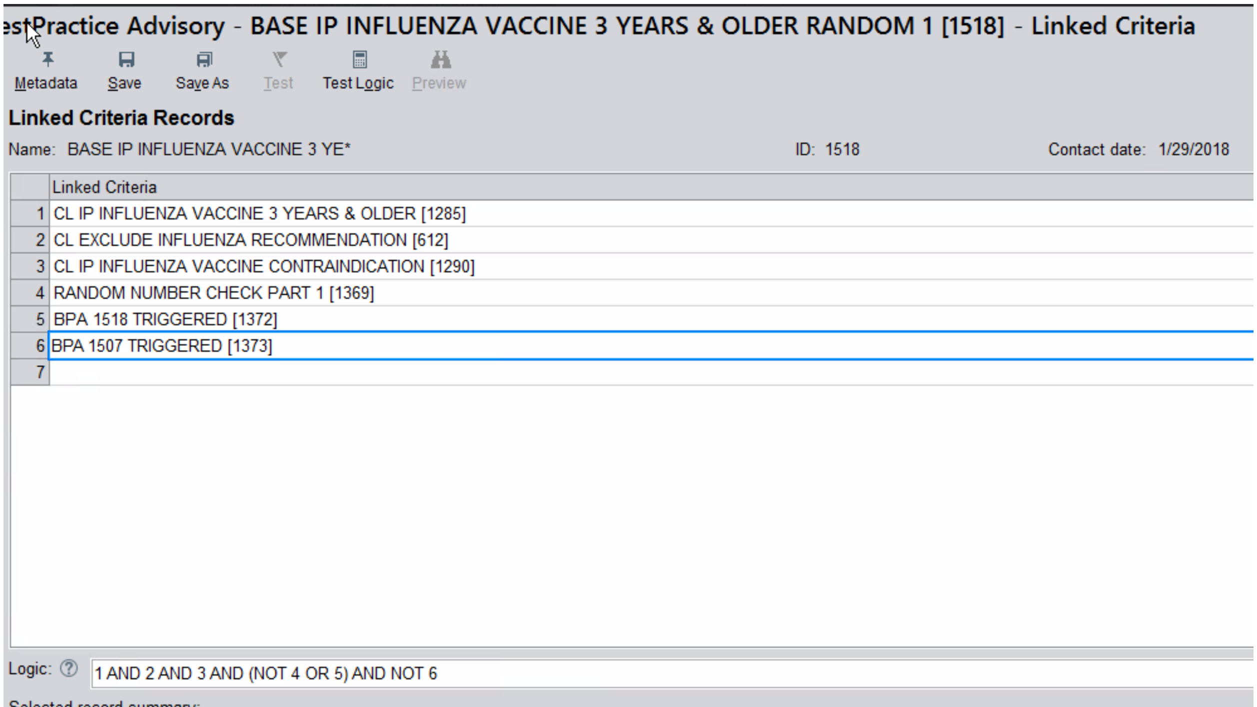


© 2020 Epic Systems Corporation.

Base Record = Base IP Influenza Vaccine 3 Years & Older Random [1518]

| Criteria Record | Sumary Logic | Explicit Logic within Criteria | Present in other Flu BPA | Other BPA |
|---|---|---|---|---|
| CL IP INFLUENZA VACCINE 3 YEARS & OLDER [1285] | Patient between 3 and 64 without Flu Vaccine Contraindication | Patient Age between 3 and 64 with no Influenza Vaccine Contraindications | Yes | BASE IP INFLUENZA VACCINE 3 YEARS & OLDER [1507] |
| CL EXCLUDE INFLUENZA RECOMMENDATION [612] | Exclude patients who already have flu vaccine | Rule within criteria to Exclude Patients who have Active Medication Order for the Flu Vaccine | Yes | BASE IP INFLUENZA VACCINE 3 YEARS & OLDER [1507] |
| CL IP INFLUENZA VACCINE CONTRAINDICATION [1290] | Exclude patients who present a contraindication or have refused the flu vaccine already | Rule within criteria to Exclude Patients with a documented flowsheet value related to Influenza that includes any of the following: Allergy to eggs or latex, age less than 6 months, completely vaccinated during current flu season, prior sensitivity to flu vaccine, patient cautioned against flu vaccine due to allergy, admitted for transplant of stem cell, solid organ, bone marrow, treatment plan is comfort/hospice, History of Guilian-Barre Syndrome after vaccine, April 2 through August (vaccine not offered), or Patient/caregiver refuses vaccine, no contraindications. | Yes | BASE IP INFLUENZA VACCINE 3 YEARS & OLDER [1507] |
| RANDOM NUMBER CHECK PART 1 [1369] | Include rule with looking for the Number 1 in a range from 1 to 2. | Rule within criteria to Include Random Number Property [208] under context BPA Locator where scenarios qualifying for the random number check. Rule looks to find a Random whole number of 1 in a range from 1 to 2. | No | N/A |
| BPA 1518 TRIGGERED [1372] | Include rule to see if this BPA has already been triggered | Rule using property **Has BPA Triggered for Patient [29001]** with BPA specified with value of yes. | No | N/A |
| BPA 1507 TRIGGERED [1373] | Include rule to see if BPA **BASE IP INFLUENZA VACCINE 3 YEARS & OLDER [1507]** has already fired | Rule using property **Has BPA Triggered for Patient [29001]** with BPA specified with value of yes to see if BPA **BASE IP INFLUENZA VACCINE 3 YEARS & OLDER [1507]** has already fired | No | N/A |
| | | | | |
| **Logic within base record** | **1 AND 2 AND 3 AND (NOT 4 OR 5) AND NOT 6** | | | |
| **Overall Logic in written text** | Vaccine Contraindication **AND** patient has not already had a flu vaccine **AND** patients does not present a contraindication or has already | | | |
| | **AND** patient does not qualify for the random number check or they have not already had this BPA fire | | | |
| | **AND** patient has not already had **BPA BASE IP INFLUENZA VACCINE 3 YEARS & OLDER [1507]** fire | | | |
| | **THEN** - trigger BPA | | | |

© 2020 Epic Systems Corporation.
